# Insights into antimicrobial resistance among long distance migratory East Canadian High Arctic light-bellied Brent geese (*Branta bernicla hrota*)

**DOI:** 10.1186/s13620-016-0072-7

**Published:** 2016-09-15

**Authors:** Austin Agnew, Juan Wang, Séamus Fanning, Stuart Bearhop, Barry J. McMahon

**Affiliations:** 1 UCD School of Agriculture & Food Science, University College Dublin, Belfield, Dublin 4 Ireland; 2 UCD-Centre for Food Safety, School of Public Health, Physiotherapy & Sports Science, University College Dublin, Belfield, Dublin 4 Ireland; 3 Institute for Global Food Security, School of Biological Sciences, Queen’s University Belfast, Stranmillis Road, Belfast, BT9 5AG Northern Ireland; 4 Centre for Ecology & Conservation, University of Exeter, Cornwall Campus, Penryn, Cornwall TR10 9FE UK

**Keywords:** Antimicrobial resistance, Wild birds, Migratory, Wildlife, Light-bellied Brent geese

## Abstract

**Background:**

Antimicrobial resistance (AMR) is the most significant threat to global public health and ascertaining the role wild birds play in the epidemiology of resistance is critically important. This study investigated the prevalence of AMR Gram-negative bacteria among long-distance migratory East Canadian High Arctic (ECHA) light-bellied Brent geese found wintering on the east coast of Ireland.

**Findings:**

In this study a number of bacterial species were isolated from cloacal swabs taken from ECHA light-bellied Brent geese. Nucleotide sequence analysis identified five species of Gram-negative bacteria; the dominant isolated species were *Pantoea spp.* (*n* = 5) followed by *Buttiauxella agrestis* (*n* = 2). Antimicrobial susceptibility disk diffusion results identified four of the *Pantoea spp.* strains, and one of the *Buttiauxella agrestis* strains resistant to amoxicillin-clavulanic acid.

**Conclusion:**

To our knowledge this is the first record of AMR bacteria isolated from long distance migratory ECHA light-bellied Brent geese. This indicates that this species may act as reservoirs and potential disseminators of resistance genes into remote natural ecosystems across their migratory range. This population of geese frequently forage (and defecate) on public amenity areas during the winter months presenting a potential human health risk.

## Findings

Antimicrobial resistance (AMR) is the greatest challenge facing global public health [1]. The current proliferation of multidrug-resistant pathogens, prevalence of resistant bacteria in the environment and dissemination of resistance genes into novel biogeographic regions is unprecedented [2].

Accurate and meaningful information relating to the dissemination of resistance genes in bacteria among wildlife is of importance in assessing the potential human health risks, and ecological impacts the ingress of these elements have on natural environments [3]. Wild birds are increasingly being studied as vectors for the transmission of resistant bacteria and the resistance genes they harbour [4, 5]. The East Canadian High Arctic (ECHA) light-bellied Brent goose (*Branta bernicla hrot*a) undertakes one of the longest migrations of any Palaearctic goose species, migrating annually from their breeding grounds in the high Canadian Arctic to Ireland in winter [6]. Their preferred food is the intertidal marine grass (*Zostera* spp), but this resource becomes exhausted in mid to late winter and the birds switch to feeding on terrestrial grasses [7]. In many parts of their range this brings them into close contact with humans as in urban areas these terrestrial grasses tend to be found in public parks and sports grounds. In this study we aimed to investigate the prevalence of clinically relevant antimicrobial resistant Gram-negative enteric bacteria carried among this population of Brent geese during their winter staging on the east coast of Ireland, and inform if these migratory wild birds are potential disseminators of resistance genes into remote natural ecosystems.

The animal trapping and handling undertaken as part of this research was carried out under permit from the British Trust for Ornithology (Permit A4656) and the Irish Medicines Board (Authorisation number AE19130/141). The project was reviewed and deemed fit by the University of Exeter Ethics Committee. A total of 66 Brent geese were caught using explosive cannon nets on North Bull Island, Dublin on the east coast of Ireland (53°22′14.92 N, 6°9′9.98 W) 31st March 2015. All birds had faecal cloacal swabs taken. Samples were placed in a cooler box and transported to the laboratory where they were stored at 4 °C prior to analyses.

Biochemical testing was conducted on all cultured isolates to test for the presence of *Escherichia coli* using indole and citrate utilisation tests. A sub-set of 16 plates shown to support good colony growth, and screened for *E. coli*, was selected for further bacterial identification using the Gram stain method. Ten isolates positively identified as Gram-negative bacterial rods following microscopic examination under an oil immersion lens were selected for DNA isolation and PCR. Following user protocol, DNA was isolated from the selected bacterial isolates using the UltraClean® Microbial DNA Isolation Kit and subsequently stored at 4 °C for downstream PCR analysis. The PCR reaction mixture was prepared for 12 samples, including one positive and one negative control, 22 μl of PCR reaction mixture was combined with 3 μl of extracted template DNA [equivalent to approx., 100 ng] for each sample. The forward and reverse bacterial primer pair sequences used in the PCR amplification process were Bakt_341F (5′-CCT ACG GGN GGC WGC AG-3′) and Bakt_805R (5′- GAC TAC HVG GGT ATC TAA TCC-3′) with an amplicon size of 464 base pair(s) (bp) [8]. The thermal cycle for PCR amplification consisted of an initial denaturation step at 95 °C for 30 s, 35 cycles of denaturation at 95 °C for 30 s, annealing at 54 °C for 1 min, extension at 68 °C for 2 min and a final elongation step at 68 °C for 2 min. A preliminary characterisation of the quality of the amplified DNA was conducted, using agarose gel electrophoresis, to establish that the required sequence had been successfully amplified for all ten PCR products along with two controls. Following electrophoresis the DNA bands were excised from the gel under UV light and prepared for sequencing using the Wizard® SV Gel and PCR Clean-Up System. The purified DNA was stored at 4 °C before same day collection and shipment for Sanger sequencing by Source BioScience Sequencing. Five microliter of primers and DNA were sent per reaction at concentrations of 3.2 pmol/μl and 1 ng/μl per 100 bp respectively. The nucleotide sequence queries for the ten bacterial samples were loaded into the National Centre for Biotechnology Information (NCBI) Basic Local Alignment Search Tool (BLAST®) [9]. A standard nucleotide BLAST was conducted using the Megablast programme BLASTN 2.2.31 [10], optimised to identify highly similar sequences. The highest Query cover and Ident percentage scores were used to determine best fit for sequence alignment. Disk diffusion antimicrobial susceptibility testing was conducted on all sequenced and identified isolates in accordance with the standardised methodology developed by the European Committee on Antimicrobial Susceptibility Testing (EUCAST), Version 5.0 [11, 12]. Isolates were tested for susceptibility to Amoxicillin-clavulanic acid (20/10 μg), Cefepime (30 μg), Imipenem (10 μg), Ciprofloxacin (5 μg) and Trimethoprim-sulfamethoxazole (1.25/23.75 μg). Inhibition zone diameters were recorded and categorised according to the EUCAST Clinical Breakpoint table v 5.0 [12].

Gram-stain tests identified a selection of Gram-negative rods (*n* = 11), Gram-negative cocci (*n* = 1), Gram-positive cocci (*n* = 1) and samples containing a mix of both Gram-negative rods and cocci (*n* = 2). Sanger sequencing successfully yielded nucleotide sequences for all ten isolated DNA samples and BLAST analysis identified five species of Gram-negative bacteria. Antimicrobial susceptibility disk diffusion results identified four of the *Pantoea spp.* strains, and one of the *Buttiauxella agrestis* strains resistant to amoxicillin-clavulanic acid. All strains were susceptible to the remaining antimicrobial compounds (Table 1).

**Table 1:** Antimicrobial disk diffusion results, bacterial species, individual bird leg ring codes, antimicrobial agents tested and zone diameter breakpoints for *Enterobacteriaceae bacterium*

		Antimicrobial Agent				
		IPM 10 03BCg	AMC 20/10 μg	SXT 23.75/1.25 μg	CIP 5 μg	FEP 30 μg
		*Enterobacteriaceae bacterium* Zone Diameter Breakpoints				
		S ≥ 22 I 16-21 R <16	S ≥19 I NA R <19	S ≥ 16 I 13-15 R < 13	S ≥ 22 I 19-21 R < 19	S ≥ 24 I 21-23 R < 21
Ring code	Species					
XK BY	*Pantoea spp.*	32 S	15 R	34 S	32 S	31 S
		33 S	15 R	34 S	30 S	34 S
		35 S	14 R	F -	32 S	33 S
JL BY	*Pantoea spp.*	34 S	16 R	34 S	32 S	34 S
		30 S	14 R	30 S	32 S	36 S
		32 S	16 R	36 S	32 S	36 S
BT RR	*Pantoea spp.*	36 S	19 S	38 S	34 S	38 S
		38 S	16 R	36 S	33 S	36 S
		37 S	17 R	38 S	34 S	37 S
KL BY	*Pantoea spp.*	34 S	17 R	34 S	32 S	35 S
		36 S	19 S	34 S	32 S	34 S
		36 S	17 R	36 S	33 S	36 S
VL BY	*Buttiauxella agrestis*	30 S	16 R	31 S	27 S	32 S
		31 S	14 R	32 S	28 S	32 S
		30 S	18 R	30 S	26 S	29 S

Abbreviations: *IPM* imipenem, *AMC* amoxicillin-clavulanic acid, *SXT* sulphamethoxazole/trimethoprim, *CIP* ciprofloxacin, *FEP* cefepime, *S* susceptible, *I* intermediate, *R* Resistant, *F* Fail

Wild birds, particularly migratory waterfowl, can travel immense distances, inhabit a wide variety of environments and may consequently have a significant epidemiological role in the dissemination of resistant bacteria and genes [13]. Non-migratory Canada Geese (*Branta canadensis*) have previously been identified as reservoirs of multi-resistant strains of *E. coli* and implicated in microbial water contamination; although non-migratory, these birds could serve to disperse bacteria between widely separated locations [14]. The finding that ECHA light-bellied Brent geese are reservoirs of resistant bacteria has direct implications related to the potential of this species to act as disseminators of resistance into remote habitats throughout their migratory range. The potential for migratory birds to carry AMR bacteria over remarkable distances to remote locations in the Arctic, a region formerly considered one of the last outposts of wilderness, has been demonstrated [15]. Sjölund et al. [15] have shown *E. coli* isolates expressing multi drug resistance to as many as eight antibiotics among Glaucous gulls in the Arctic. This study also found a juvenile Western sandpiper sampled on the Siberian tundra had resistance to cefadroxil, cefuroxime, and cefpodoxime, a resistance pattern commonly observed in clinical isolates, supporting the theory of introduction by migration and subsequent bacterial transfer between birds. The potential for wild birds to act as vectors for the transmission of clinically relevant resistance genes is substantiated by the discovery of gulls harbouring the same CTX-M types dominant among human isolates in the same area [4, 16]. Hernandez et al. [17] found the carriage rate of ESBL-producing bacteria among Franklin’s gulls in central parts of Chile to be twice as high as those found among the human population in the same area, but the gulls were also found to share sequence types from clinical samples in central Canada, a known nesting place for the birds. These findings contribute to the accumulating evidence supporting the dissemination of resistance by migratory birds, and the reciprocal transmission of resistance determinants between humans and wild birds [18].

It seems likely that the greatest exposure to environmental sources of antibiotics and resistant bacteria the light-bellied Brent geese encounter throughout their migration is during the winter in Ireland, where they experience the most intense contact with anthropogenic-influenced habitats. Previous studies have isolated AMR *E. coli* from herring gulls (*Larus argentatus*) sampled in Howth harbour located within 10 km of North Bull Island [19–21]. ECHA light-bellied Brent geese migrate north from Ireland during the spring and stage in western Iceland [7], before continuing their journey to breed in the Canadian Arctic [22], satellite tracking has also identified a number of staging grounds in east Greenland [23]. AMR found among this population of Brent geese identifies them as potential disseminators of resistant bacteria, and the genetic resistance determinants they possess, into various ecosystems throughout their range.

Resistance found among the *Pantoea* species isolates in this research is notable as multiple species groups within *Pantoea* are viewed as opportunistic pathogens [24]. Perhaps, the greatest zoonotic potential the birds sampled in this research present may be through their use of amenity grasslands. Faecal shedding of resistant bacteria and the persistence of such organisms in the environment may pose a health threat to humans [25]. A study by Benson [26] identified 60 inland sites used by light-bellied Brent geese as winter feeding grounds in Dublin, these include playing pitches, public parks, golf clubs and municipal green spaces in densely populated areas. The large amount of faeces resulting from congregating flocks on amenity grassland could present a possible health risk.

Future research, in conjunction with the findings here, could help elucidate the persistence of resistant bacteria carried by the birds throughout their range at different times of the year, accurately appraise their ability to act as vectors for the dissemination of resistance and define where resistant bacteria is acquired. The enrichment of environmental bacteria with genetic elements containing resistant genes, the dynamic nature of prokaryotic genomes, and ease with which resistance determinants can be shared among commensal and pathogenic bacteria, conspire to present a threat to both human and animal health. This research has identified ECHA light-bellied Brent geese as reservoirs of resistant bacteria and potential disseminators of resistance genes into remote natural habitats in Iceland, Greenland and the Canadian Arctic. The findings in this study add to the accumulating evidence that wild migratory birds are disseminators of resistant bacteria and can play an important role in the epidemiology of resistance. AMR is a global health concern and reaches far beyond clinical settings, understanding the role wildlife plays, particularly migratory birds, is critically important in designing practicable and effectual mitigation measures to address this problem for the future.

## Abbreviations

AMR: Antimicrobial resistance
ECHA: East Canadian High Arctic
EUCAST: European committee on antimicrobial susceptibility testing
ESBL: Extended-spectrum β-lactamase

## References

[1] Laxminarayan R, Duse A, Wattal C, Zaidi AKM, Wertheim HFL, Sumpradit N (2013). Antibiotic resistance - the need for global solutions. Lancet Infect Dis.

[2] Levy SB, Marshall B (2004). Antibacterial resistance worldwide: causes, challenges and responses. Nat Med.

[3] Chee-Sanford JC, Mackie RI, Koike S, Krapac IG, Lin Y-F, Yannarell AC (2009). Fate and transport of antibiotic residues and antibiotic resistance genes following land application of manure waste. J Environ Qual.

[4] Bonnedahl J, Drobni P, Johansson A, Hernandez J, Melhus A, Stedt J (2010). Characterization, and comparison, of human clinical and black-headed gull (*Larus ridibundus*) extended-spectrum β-lactamase-producing bacterial isolates from Kalmar, on the southeast coast of Sweden. J Antimicrob Chemother.

[5] Hernandez J, Bonnedahl J, Eliasson I, Wallensten A, Comstedt P, Johansson A (2010). Globally disseminated human pathogenic *Escherichia coli* of O25b-ST131 clone, harbouring blaCTX-M-15, found in Glaucous-winged gull at remote Commander Islands, Russia. Environ Microbiol Rep.

[6] Robinson JA, Colhoun K, Gudmundsson GA, Boertman OJ, Merne M, O’Briain M (2004). Light-Bellied Brent Goose *Branta Bernicla Hrota* (East Canadian High Arctic Population) in Canada, Ireland, Iceland, France, Greenland, Scotland, Wales, England, the Channel Islands and Spain: 1960/61-1999/2000.

[7] Inger R, Gudmundsson GA, Ruxton GD, Newton J, Colhoun K, Auhage S (2008). Habitat utilisation during staging affects body condition in a long distance migrant, *Branta bernicla hrota*: potential impacts on fitness?. J Avian Biol.

[8] Herlemann DP, Labrenz M, Jürgens K, Bertilsson S, Waniek JJ, Andersson AF (2011). Transitions in bacterial communities along the 2000 km salinity gradient of the Baltic Sea. ISME J.

[9] Johnson M, Zaretskaya I, Raytselis Y, Merezhuk Y, Mcginnis S, Madden TL (2008). NCBI BLAST: a better web interface. Nucleic Acids Res.

[10] Zhang Z, Schwartz S, Wagner L, Miller W (2000). A Greedy Algorithm for Aligning DNA Sequences. J Comput Biol.

[11] EUCAST. European Committee on Antimicrobial Susceptibility Testing. Disk Diffusion Test Methodology, version 5.0, 26 January 2015, Available at: http://www.eucast.org/ast_of_bacteria/disk_diffusion_methodology/. (Accessed 09 July 2015).

[12] EUCAST. European Committee on Antimicrobial Susceptibility Testing. Breakpoint tables for interpretation of MICs and zone diameters. Version 5.0, 2015. Available at: http://www.eucast.org/clinical_breakpoints/. (Accessed 09 July 2015).

[13] Allen HK, Donato J, Wang HH, Cloud-Hansen KA, Davies J, Handelsman J (2010). Call of the wild: antibiotic resistance genes in natural environments. Nat Rev Microbiol.

[14] Cole D, Drum DJ, Stallknecht DE, White DG, Lee MD, Ayers S (2005). Free-living Canada Geese and Antimicrobial Resistance. Emerg Infect Dis.

[15] Sjölund M, Bonnedahl J, Hernandez J, Bengtsson S, Cederbrant G, Pinhassi J (2008). Dissemination of multidrug-resistant bacteria into the Arctic. Emerg Infect Dis.

[16] Simões RR, Poirel L, Costa PMD, Nordmann P (2010). Seagulls and Beaches as Reservoirs for Multidrug-Resistant *Escherichia coli*. Emerg Infect Dis.

[17] Hernandez J, Johansson A, Stedt J, Bengtsson S, Porczak A, Granholm S (2013). Characterization and Comparison of Extended-Spectrum β-Lactamase (ESBL) Resistance Genotypes and Population Structure of *Escherichia coli* Isolated from Franklin's Gulls (*Leucophaeus pipixcan*) and Humans in Chile. PLoS ONE.

[18] Bonnedahl J, Järhult JD (2014). Antibiotic resistance in wild birds. Ups J Med Sci.

[19] Smith S, Wang J, Fanning S, Mcmahon BJ (2014). Antimicrobial resistant bacteria in wild mammals and birds: a coincidence or cause for concern?. Ir Vet J.

[20] Carroll D, Wang J, Fanning S, Mcmahon BJ (2015). Antimicrobial resistance in wildlife: implications for public health. Zoonoses Public Health.

[21] Stedt J, Bonnedahl J, Hernandez J, Waldenström J, Mcmahon BJ, Tolf C (2015). Carriage of CTX-M type extended spectrum β-lactamases (ESBLs) in gulls across Europe. Acta Vet Scand.

[22] Gudmundsson GA, Benvenuti S, Alerstam T, Papi F, Lilliendahl K, Akesson S (1995). Examining the limits of flight and orientation performance: satellite tracking of Brent geese migrating across the Greenland ice-cap. Proc R Soc Lond B Biol Sci.

[23] Robinson JA, Gudmundsson GA, Clausen P, Boere GC, Galbraith CA, Stroud DA (2006). Flyways of the East Canadian High Arctic Light-bellied Brent Goose *Branta bernicla hrota*: results of a satellite telemetry study. Waterbirds around the world.

[24] Nadarasah G, Stavrinides J (2014). Quantitative evaluation of the host-colonizing capabilities of the enteric bacterium *Pantoea* using plant and insect hosts. Microbiology.

[25] Fallacara DM, Monahan CM, Morishita TY, Wack RF (2001). Fecal shedding and antimicrobial susceptibility of selected bacterial pathogens and a survey of intestinal parasites in free-living waterfowl. Avian Dis.

[26] Benson L (2009). Use of inland feeding sites by Light-bellied Brent geese in Dublin 2008–2009: a new conservation concern. Ir Birds.

